# Physical transition from professional MMA to tactical firefighting: case study

**DOI:** 10.1080/15502783.2025.2550157

**Published:** 2025-08-25

**Authors:** Cole Smeester, Gabriel J. Sanders, Corey A. Peacock, Jose Antonio

**Affiliations:** a Nova Southeastern University, Department of Health and Human Performance, Davie, FL, USA; b University of Cincinnati, Exercise Science, Cincinnati, OH, USA

**Keywords:** Resistance training, body composition, performance, tactical

## Abstract

**Background:**

Firefighting is a physically and mentally demanding line of work, requiring heightened strength, conditioning, and cognition to perform job-specific tasks safely and effectively. To prepare for these demands, recruits undergo the Emergency Physical Ability Test (EPAT), a simulation-based physical assessment used in firefighter selection. This case study evaluated the physiological and physical performance changes in a retired professional heavyweight martial arts (MMA) athlete transitioning into a career as a firefighter.

**Methods:**

The subject (age: 32 years; height: 203.2 cm) completed an 8-week training program with four weighted-resistance and two conditioning sessions per week. The program began with hypertrophy-focused training and progressed to power and strength development. Conditioning included one sprinting (100 m, 200 m, 400 m) and one long-distance (5 km) session on nonresistance days. Resistance training featured sled pushes/pulls, weighted carries, stair climbs, and weight room exercises to replicate firefighting demands and build strength. EPAT performance was assessed by timing the full simulation course. One-mile runs were timed outdoors on a flat surface. Body composition was measured using a multi-frequency BIA device (InBody 270) following manufacturer instructions. All data was collected at baseline and post-training, with percentage change scores calculated using Microsoft Excel.

**Results:**

Following the 8-week intervention, the participant’s body weight increased from 120.7 kg to 129.6 kg, representing a 7.4 percent increase. Skeletal muscle mass increased from 58.0 kg to 62.4 kg, a 7.6 percent gain, while body fat percentage remained constant at 17 percent. Physical performance also improved, with EPAT time decreasing from 7:27 to 7:00, reflecting a 6.0 percent improvement. Additionally, the one-mile run time decreased from 12:00 to 11:25, a 4.9 percent improvement in aerobic capacity.

**Conclusion:**

An 8-week tactical training program utilizing conjugate methodology with varied resistance exercises every four weeks resulted in increased skeletal muscle mass and improved occupational performance. This was evidenced by faster EPAT and one-mile run times and improved body composition. Despite a 7.4% weight gain, the subject increased cardiovascular and muscular ability, supporting the benefit of muscle mass and strength on cardiovascular performance. As the subject transitioned from professional MMA to firefighting, the training demands changed from high-volume fight preparation to occupationally specific strength and conditioning.

